# Neighbourhood, Built Environment and Children’s Outdoor Play Spaces in Urban Ghana: Review of Policies and Challenges

**DOI:** 10.1016/j.landurbplan.2021.104288

**Published:** 2021-11-05

**Authors:** Dina Adjei-Boadi, Samuel Agyei-Mensah, Gary Adamkiewicz, Judith I Rodriguez, Emily Gemmell, Majid Ezzati, Jill Baumgartner, George Owusu

**Affiliations:** Department of Geography and Resource Development, University of Ghana, Legon, Accra, Ghana; Department of Geography and Resource Development, University of Ghana, Legon, Accra, Ghana; Harvard School of Public Health, Harvard University, USA; Graduate School of Design (GSD), Harvard University, USA; School of Population and Public Health, University of British Columbia, Canada; School of Public Health, Imperial College London, UK/Regional Institute of Population Studies (RIPS), University of Ghana, Legon, Accra, Ghana; Department of Epidemiology, Biostatistics, and Occupational Health McGill University, Canada; Institute of Statistical, Social & Economic Research (ISSER), University of Ghana, Legon, Accra

**Keywords:** play, playability, outdoor play space, children play areas

## Abstract

Although a great deal of research work has been done by social scientists on walkability and playability, the focus to a large extent has been on the global north. Research work on the urban built environment and children’s play has not engaged Africa in general and Ghana in particular. More importantly, there is limited evidence of policies in terms of community-based practices and governmental policies and programmes for the promotion of play. The limited effort in promoting physical activities have to a large extent focused on walkability, yet evidence to date indicates that walking only constitutes a small proportion of the physical activities of children. This is against the backdrop of growing urbanization and the increasing reported incidence of sedentary lifestyles, less physical activity and obesity among children and the youth. Our main objective in this paper is to contribute to the literature on Ghana, and by extension Sub-Saharan Africa, by examining the extent to which playability features in city and national policies and strategies in urban Ghana. We conclude that while there is dearth of public policies on children’s play, with the situation in communities compounded by weak city government capacity to plan, implement and enforce development control to protect open spaces for children’s play and recreational purposes. The paper recommends a change in policy and practice on creating spaces in urban built-environments for children’s play in urban Ghana.

## Introduction

Across the world, it is generally accepted that play is critical to children’s emotional and physical development, health, and general well-being (Clements, 2004; Lester & Russell, 2008; Janssen & King, 2015; Lambert et al., 2019).^1^ Consequently, children’s ability to play is recognized under Article 31 of the United Nations Convention on the Rights of the Child (UNCRC) and Article 12 of the African Charter on the Rights and Welfare of the Child (ACRWC). Both articles of these international conventions recognized the right of every child “to rest and leisure, to engage in play and recreational activities appropriate to the age of the child and to participate freely in cultural life and the arts”.^2^

Also, playgrounds and public open spaces for children’s play have gained international recognition in recent years reflected in its inclusion in the Sustainable Development Goals (SDGs). Target 11.7 of SDG 11 on sustainable cities states that: “By 2030, provide universal access to safe, inclusive and accessible, green and public spaces, in particular for women and children, older persons and persons with disabilities” (UN General Assembly, 2015, 22). This means that both developed and developing countries are tasked with the responsibility of ensuring that their populations (including children) have access to parks, open and green spaces, and playgrounds (Shoari et al., 2021) because they aid in not only good health but also provide emotional and psychological benefits like reduction of depression and stress and enhance self-esteem (Floriani & Kennedy, 2008; Roe et al., 2013; Beyer et al., 2014; Aspinall et al., 2015; Feng et al., 2017; Tillmann et al., 2018). Randomized and quasi-randomized studies show that the greening of vacant urban land for play may contribute to reduced crime and violence (Branas et al., 2011; Garvin et al., 2012; Macdonald et al., 2021), build social networks and cohesion (Veitch et al., 2010), reduce the likelihood and risk of overweight and obesity (Rodearmel et al., 2007; Wolch et al., 2011; Dadvand et al., 2014), increase physical activity (Raney et al., 2019) and improve mental health (Liu et al., 2019). Playgrounds and public open spaces also serve as places of relaxation and contact with the natural environment (Veitch, Salmon & Ball, 2010).

However, Janssen & King (2015) and Lambert et al. (2019) have argued that children’s play activities are shaped by the environment and the perceptions of the environment in which they live. According to Lambert et al., (2019, 2), “physical activity behaviours are intimately influenced by characteristics of the environment including neighbourhood design (e.g., traffic, pedestrian facilities, aesthetics), as well as how these aspects of the environment are perceived”. Janssen & King (2015) have noted that research on the physical activities of adults largely focused on the neighbourhood walkability as a function of how well streets are connected, the density of people and places, diversity of land use, and presence of pedestrian infrastructure. They add that while walking is the primary source of physical activity for adults, for children, neighbourhood playability (presence of yards, playgrounds, undeveloped green spaces, cul-de-sacs, very low traffic streets, and trails) is the key determinant of their physical activities.^3^ This is because walking accounts for only a small proportion of the physical activities of children (Owen, 2014; Faulkner et al., 2009; Janssen & King, 2015).

Gender also has a key role to play despite the different factors that influence children’s outdoor play activities (Collier & Hines, 1995; Carver et al., 2010; Mitra et al., 2014; Alanazi et al., 2020). Gender differences determine the choice of play activity and behaviour and this is attributed to sexual hormones (Pellegrini & Smith,1998; Bjorklund & Pellegrini, 2000). A lot of studies report that boys engage in active and structured outdoor play than girls who prefer to play in smaller groups near their homes (Carver et al., 2008; Larson, Green, & Cordell, 2011; Bichara, 2012; Leung & Loo, 2017). Nonetheless, similar to boys, evidence exists of the positive association between girls’ outdoor play and their health. According to Piccininni et al. (2018), girls who spend 30 minutes engaging in outdoor play every week have a lower prevalence of psychosomatic symptoms, compared with girls who do not.

Although a great deal of research work has been done by social scientists on walkability and playability, the focus to a large extent has been on the global north, especially Europe, North America, and Australia (Barr et al., 2020). Indeed, Lambert et al. (2019) in a detailed systematic literature review of the relationship between the neighbourhood, built-environment, and time spent by children in outdoor play did not identify a single reference from Africa. Research work on the built environment and children’s play has not engaged Africa in general and Ghana in particular, compared with the global north.

However, it is gratifying to note that some recent studies on physical activities in cities in Sub-Saharan Africa have focused on walkability which as noted earlier relates more to adults (see Oyeyemi et al., 2019a, 2019b; Barr et al., 2020; Oviedo et al., 2021). In addition, we have seen in recent year’s attempts to include a limited number of countries in Africa, specifically Ghana, Kenya, and South Africa, in the global Report Card on Physical Activity for Children and Youth to establish national baseline data on physical activity (PA) and PA-enabling environments (Ocansey et al., 2014). Despite the vast knowledge of how important outdoor play is to the growth and development of children including their overall mental and social health (Fjøtoft, 2001; Waller, 2006; Ginsburg & Kingery, 2007; Veitch et al., 2010; Liu et al., 2019), one needs to ask why the limited attention to this matter in terms of research and more importantly policy terms. Specifically, for Ghana, to the best of our knowledge, no academic work has been done on the urban built environment and children’s outdoor playability. Aryeetey (2011) assessed features of the built environment using a street-level audit of East Legon, a high-income neighbourhood in Accra. He identified barriers to neighborhood walkability which included the absence of sidewalks, poor access to sidewalks, and poor conditions of sidewalks, but did not assess children’s outdoor play activities.

This paper aims to contribute to the limited literature on playability for countries in Sub-Saharan Africa. It examines the extent to which playability features in city and national policies and strategies in Ghana, and illustrates the limited opportunities for children’s outdoor play spaces in the Greater Accra Metropolitan Area (GAMA), the largest metropolitan region in the country. This is against the backdrop of rapid urbanization across Ghana and the rest of Sub-Saharan Africa with expected declines in physical activity and lifestyle changes (Barr et al., 2020). The paper is structured into seven sections. After the introduction, the next section examines the definition of children’s play followed by a section on the study’s research methodology. The paper then reviews relevant policy documents on urban development and children’s play and then a section on the institutional framework for land use and children’s play spaces. Next, the paper looks at the built environment of GAMA and play spaces, and ends with a conclusion.

## Defining children’s play

Play is well-recognized as a very key component of a child’s life, and the activities that constitute children’s play can evolve over time (Gill, 2021) and may be more or less emphasized in different settings depending on the attitudes to children and to play, which are affected by cultural, socio-economic, and environmental circumstances. Many researchers noted the different aspects and nature of play across different settings (Webb & Associates, 1999; Cole-Hamilton, Harrop & Street, 2002), and hence, the difficulty in defining it. For instance, children running around the compound of a house, playing football, or dressing up their dolls – all constitute play. This leads Garvey and Lloyd (1990) to conclude that although the “central notion of play seems clear enough, the fringes of the concept are fuzzy”.

The difficulty in defining play is exacerbated when it is compared to terms such as ‘recreation’ and ‘leisure’. This is because these terms share some similar characteristics (including the involvement of some level of physical activities) and outcomes such as reduction in stress and improved health. Nevertheless, in broad terms, McLean & Hurd (2012) have noted that play can be distinguished from leisure and recreation, although the lines of distinctions can be blurred. While play can be broadly defined as an activity that is self-motivated and undertaken for intrinsic purposes, leisure refers generally to how an individual uses his or her free time (Henderson, 2010; McLean & Hurd, 2012). On the other hand, recreation is defined as the activities undertaken during one’s free time and these activities are generally voluntary and for pleasure purposes (Henderson, 2010).^4^

Given the difficulty in defining play in precise terms, many definitions tend to focus on the characteristics of play. According to the Scottish Government (2013, 12) ‘play is freely chosen, personally directed, intrinsically motivated behaviour that actively engages the child.’ This means that play “is performed for no external goal or reward, and is a fundamental and integral part of healthy development - not only for individual children but also for the society in which they live” (Welsh Assembly Government, 2002, 3). Bartlett (1999, 68) also defines play as children getting involved ‘passionately in their surroundings through exploration, manipulation, physical exuberance, experimentation, and pretense, either alone or with others ‘. Bartlett adds further that play is a “basic human drive” very key to the development process of children and supports this statement with the fact that neuropsychology and psycho-pharmacology information iterated that “distinct changes in the brain occur as a result of play”.

Garvey (1977) believed that play is associated with pleasure and fun, and the player has an active role in the entire process. Children spend most of their day playing actively with their play evolving from noisy play with friends to exploring and imaginative play alone (Dali, 2010). Play can therefore be considered as “children’s unique way of learning about their world, but also their way of learning about themselves and how they fit into their world” (Childhood Education, 2002), and this can only be possible depending on the kind of play features within the immediate environment (Fjortoft & Sageie, 2000). Play in different environments has different impacts on children in terms of cognitive and social development (Youell, 2008; Fjortoft & Sageie, 2000; Bartlett, 1999). Children tend to define their interests and types of activity to engage in when playing, and whether they are successful or not is determined by them. According to Boreham and Riddoch (2001), children view play as their work and they take it very seriously.

In sum, beyond gender, play is strongly influenced by the settings of the built and natural environments, or what may be termed as playability. According to Han et al. (2018), “playability” is defined as how friendly environments are for children’s outdoor play and independent mobility. In other words, playability relates more to the built and natural environmental setting (including the policies shaping this) whereas play is the activities undertaken in the setting. Consequently, several interrelated factors (access, safety and security, aesthetic, etc) contribute to either enhance or deter playability and the decisions of children selecting where to play.

## Relationship between play activities, culture and gender

Play like every human activity is also affected by cultural values. As such different cultures and cultural values about childhood in general see and assess the importance of play differently (Gosso & Carvalho, 2013). Some of the cultural norms have an effect on children’s play within the communities. Many of the play activities in Ghana such as hide and seek, hopscotch, cricket, and many others are structured in different cultures and modified to create local versions and children use local resources to replace the original play instrument during play. Equally interesting are the gender dimensions of play (see Table 1). For instance, in the Ghanaian context girls may experience parental or caregiver restrictions in participating in play activities that are often perceived as reserved for boys although this view is changing (see Křen et al., 2012; Nogueira et al., 2013). This also applies to “ampe”, a common children’s local play in Ghana, which is traditionally considered a play activity for girls. Skipping rope is a play activity more commonly observed among girls although some boys also engage in it though to a more limited extent.

Gender differences in the nature and type of play also cut across cultures. As such most games or play activities are gendered and clearly defined in most of our communities and this can be attributed to social identity (Bichara et al., 2012). Boys engage in any play activity that interests them, occupy larger space and can play further away from home but girls are often more restricted in the type of play activity and space and are expected to play closer to home (Goldstein, 1995; Bichara et al., 2012; Gosso et al., 2014; Leung & Loo, 2017). This is because they can be called at any time by their parents to do house chores like fetching water and washing dishes. Some physical activities are socially and culturally determined so girls should not be seen or are restricted from playing certain games such as football and a boy may be ridiculed if seen playing ‘ampe’. Most girls tend to engage in non-contact activities such as walking, dancing and skipping while boys normally engage in team and contact sports like football (Grieser et al., 2006; Prusak & Darst, 2002).

## Research methodology

We undertook a content analysis of several national policies and other strategic documents for Ghana. This analysis paid particular attention to the extent to which children’s play and recreational activities feature in terms of policy goals, objectives, and strategies in the policy documents selected. These policies and strategic documents are the Revised National Health Policy, 2020; National Urban Policy Framework (NUPF) and Action Plan, 2012; National Spatial Development Framework, 2015-2035; National Housing Policy, 2015; National Youth Policy, 2010; Gender and Children, Child and Family Welfare Policy, 2015; Ghana Building Code, 2018; and Children’s Act 1998 (Act 560). In addition, we reviewed the content of the medium-term development policy framework of the state/central government and those of the city governments or municipalities within the Greater Accra Metropolitan Area (GAMA). These medium-term development plans of the municipalities tend to be similar to that of the state as they are developed based on the guidelines provided by the National Development Planning Commission (NDPC), the apex planning body in Ghana. In addition, these municipalities operate within a fairly similar environment, that is, GAMA, the largest and densest built environment in Ghana.

We identified and listed policy documents that have the potential to impact play opportunities in Ghana. A lot of factors were taken into consideration in the preliminary listing of these policy documents some of which include the built environment, housing, issues concerning children in terms of individual safety and community safety, health, recreation, education, child right and protection, discrimination and equity. It was impossible to analyse all policy documents in these areas so it was agreed that the focus should be on policies that are directly related to children and general policies that can impact play opportunities.

Electronic copies of the policy documents were gathered from searches from different sources. This was predominantly from websites of various government departments and agencies and municipalities. After downloading the policy documents from various government websites, analyses of all the policy documents were done by identifying the aims and priorities of each policy, references to urban development, play and recreation, and its potential effect on play opportunities available to children. The process included reading each document thoroughly and searching for keywords like ‘play’ and ‘recreational activities’, summarizing each policy document and then identifying key areas that relate to children and play. For the policies on children, keywords searched included ‘play’ (play as an activity), or ‘recreation’ or ‘leisure’ and ‘activity’ or ‘activities’ and ‘space’. In addition, the reference searched include ‘child’ or ‘children’ and ‘youth’. Youth is officially defined as ages 15 to 35 years in Ghana, we included the term in our search since ages 15 to 17 years are also classified or defined as children. A grid table was drawn with all the policies written in one column and comments in another column. All page numbers or section numbers that have references to play or any of the keywords searched in that document, was noted down in the comment column. If there are no references to play at all, or any of the keywords searched, it is also noted in the comment column. The searches and extraction of documents were initially done by two authors/researchers. Afterwards, other researchers also reviewed the extraction process for purposes of validation.

Based on these searches, the policy documents were selected and analyzed according to the extent to which their policy goals, objectives, and strategies focused on children, play and recreation. In particular, our content analyses of the policy documents paid attention to references to children’s play. As such the content analyses of the policies were classified into three categories, that is, those that made reference to ‘children’, those that made reference to ‘recreational activities’ and finally those that made no mention of either play or recreational activities.

## Review of policy documents

Based on the search strategy above, 15 government policy and strategy documents were identified. However, further scrutiny of the 15 government policy and strategy documents led to the elimination of five policy documents which were found to have very little to do with play and playability, or were outdated. For example, the researchers identified and included the Zoning Guidelines and Planning Standards, 2011, as part of the 15 government policy and strategy documents. However, this document was dropped as it was outdated and superseded by the Ghana Building Code, 2018. In other words, the review focused on existing national or municipal policy documents which could impact on play opportunities. We, therefore, focused on the 10 documents captured in Table 2, which met our criteria for review. In all the 10 policy documents analyzed for this research, six documents were directly related to children. The table provides a summary of the key policy documents reviewed and their assessment along the three categories of children, play and recreation. It needs to be stressed that in broad terms, the last decade has witnessed an increasing pro-activeness of the Ghanaian state toward urban development, after decades of an attitude of ambivalence. This is reflected in the formulation of several policies either directly and indirectly towards addressing the challenges of urbanization. Most of the policy documents identified were from 2010 and have a direct and indirect focus on urban development.

Table 2 indicates the ten policy documents analyzed, only one document, the Child and Family Welfare Policy, 2015, focused exclusively on children without any reference to play and recreation. Four documents, namely the National Housing Policy, 2015; National Urban Policy Framework and Action Plan, 2012; National Spatial Development Framework, 2015-2035 and National Health Policy, 2020, provide policy goals and strategies in promoting spaces within urban communities for recreational purposes without specific reference to children. Also, four other documents have some focus on children’s recreation without the use of the term ‘play’, namely the Children’s Act of 1998 (Act 560); National Youth Policy, 2012; National Medium-Term Development Framework and the versions adopted by the municipalities within GAMA. Of all the documents reviewed, it is only the Ghana Building Code, 2018, which has specific provisions for children’s play and recreation with respect to the construction of shopping malls, schools, and open spaces within communities.

The Children’s Act (Amended) 2016 (Act 937) and Child and Family Welfare Policy, 2015, are the most directly related to children. However, both documents did not explicitly mention play or even use the word play, rather these two policies largely focused on child protection, prevention of abuse, and the general well-being of children. However, Act 937 (Amended) provides that every child has a right to social activity and that no person shall deprive a child of the right to participate in sports, or to participate in positive cultural, artistic or other recreational activities. Other policy documents, namely the National Youth Policy, 2010; National Urban Policy Framework & Action Plan, 2012; National Housing Policy, 2015 and; National Spatial Development Framework, 2015-2035, advocate for policy measurement to promote recreation and positive leisure activities through the provision of playgrounds and, green and open spaces in communities. In particular, the Youth and Sports Policy, 2010 which covers older children, calls for the development of new and upgrading of the quality of existing sports and recreational infrastructure throughout the country.

The 2018 Ghana Building Code (GBC) is the only policy document that mentioned play areas for children and further distinguished between different open spaces and how it should be designed for recreational purposes. The GBC GS1207 (Ghana Building Code, Ghana Standard 1207) of 2018 provides standards for the smooth operation and construction of residential and non-residential buildings for a safer built environment. In particular, zoning guidelines and planning standards of the Code make provision for Public Open Spaces (POS) for both formal and informal or casual recreation purposes such as parks and gardens, play areas for children, and other land uses within communities. The guideline further makes provision for children playing off-street by ensuring that all residential dwellings have small areas, open spaces or yards for children to play off the street as well as outdoor and indoor recreational facilities. It also spells out the standards for the construction of playgrounds for children (including the size, choice of location and materials to be used) in shopping malls, schools and communities (GSA, 2018). It, however, remains to be seen how the GBC will shape land use and children play spaces in urban Ghana.

In general, our review indicates that there is a policy vacuum on measures and strategies that advocate for children play and play spaces in urban Ghana. Writing on the global 2014 Report Card on Physical Activity for Children and Youth, by Active Healthy Kids Global Alliance’s “Global Matrix 3.0” in which Ghana participated for the first time, Ocansey et al. (2014, 58) concluded that there is limited evidence on physical activity levels of children and youth and less information about community-based practices or governmental programmes for the promotion of physical activity.

Indeed, as Table 3 indicates, the country scored a weak grade of D (21-40%) in 2014 and 2016 and improved marginally in 2018 with a C grade (41-60%) for overall physical activity levels among children and youth. The relatively low grade for overall physical activity among children and the youth in Ghana is buttressed by the low scores of D obtained for school infrastructure, policies and programmes; community and the built environment, and government strategies and investments (see Table 3). The results for Ghana from the 2018 global Report Card on Physical Activity for Children and Youth as contained in Table 3 are similar to grades or scores from Botswana, Ethiopia, Nigeria, South Africa and Zimbabwe (Abdeta et al, 2018; Akinroye et al., 2018; Draper et al., 2018; Manyanga et al., 2018). This shows that children and youth in other African countries are also recording low participation in physical activities and are not meeting the daily recommended physical activity requirement. Similar to Ghana, the low score of grade D was obtained for policy and infrastructure-related indicators, namely school infrastructure, policies and programmes; community and the built environment and; government strategies and investments in physical activities for children and the youth for all the African countries which participated in the survey. The overall effect of the poor policy regime for promoting and enhancing children’s play is most likely the reason for the weak/poor scores for physical activity levels among children and the youth.

These findings are consistent with the Ghana Global School-based Student Health Survey (GSHS), 2007 and 2012, which focused on children at the basic school level between ages 13 and 15 (see Table 4). For instance, Nyawornota et al. (2018) note that the 80 minutes per week of physical education in schools mandated by the Ghana Education Service is hardly being adhered to, hence, the low physical activity among children. It is therefore not surprising that as shown in Table 4 the percentage of students who were physically active for a total of at least 60 minutes per day during the past 7 days declined only marginally – 16.3% in 2007 to 16% in 2012. In the case of girls, there was no decline at all for the period. It needs to be stressed that the low scores and the high proportion of children and youth not engaged in physical activity in both the Global School-based Student Health Survey and Ghana Report Card on Physical Activity for Children and Youth are likely to be even lower in urban centers, especially large metropolitan areas like GAMA where there are limited opportunities for physical activities and high prevalence of car-dependent lifestyle.

## Institutional framework for land use and children’s outdoor play spaces in Ghana

Although there is a gap in policy terms with respect to children play, there is a multiplicity of state agencies involved in children’s welfare, including the Ministry of Gender, Children and Social Protection, Ministry of Education/Ghana Education Service, Ministry of Health, Ministry of Youth & Sports, etc. A key question requiring attention is: how do the policy goals and strategies outlined in the national policy documents such as the GBC, 2018, translate at the local and neighbourhoods of Ghanaian cities with respect to children’s outdoor play activities? This question can be addressed in the existing institutional and legal framework on urban land use in Ghana.

In Ghana, city governments referred to as Municipal and Metropolitan Assemblies (MMAs) are the main machinery for the implementation, monitoring, and evaluation of action plans at the city and community levels (MGC&SP, 2015).^5^ The existing institutional and legal framework mandates the MMAs or city governments as the main agents of local development in Ghana, including land use planning and the development and maintenance of open and green spaces for children’s play and recreation. However, city governments are generally weak both in terms of their capacity and the political will to plan, implement and enforce development control to protect open spaces for children’s play and recreational purposes (Doan & Oduro, 2011; Owusu, 2013; World Bank, 2015).

A key challenge is the existing land management system in Ghana within the context of the weak capacity of the main institutions, MMAs, for planning and zoning, and the enforcement of development controls. While land use planning and development planning in general at the city and local levels remain a public function, land for development largely remains in the hands of customary institutions (families, clans and chiefs). According to Owusu et al. (2012), due to weak capacity, city governments are unable to effectively coordinate, communicate and harmonize their land use planning efforts with customary landholders resulting in a situation where chiefs and other private landowners dispose of their lands for purposes other than what they have been zoned for by planners. In a metropolitan area such as GAMA where there is intense competition for land for residential and commercial uses and where the planning authority to enforce development control, open and green spaces for children play and recreation become spaces for encroachment and even sacrificed for other land uses. A typical example is the former Olympic Park in GAMA now the National Theatre and the Lotteries Park which has been transformed into a car park.

A conclusion in the government’s own National Medium-Term Development Policy Framework (NMTDPF), An Agenda for Jobs: Creating Prosperity and Equal Opportunity for All, 2018-2021 is worthy to note. This key national policy document concludes that “there are inadequate policies and programmes, institutions and facilities to deliberately incorporate recreation in the way of life of the people” (GoG/NDPC (2018, 60). It adds that to promote sports and recreation and, by extension, children’s play there is the need to address several constraints including inadequate and poor sports, recreational facilities; poor maintenance of existing facilities; encroachment by developers on designated sports, and recreational lands; absence of disability, child- and aged-friendly facilities; limited community-level sports and recreational activities, among others.

## GAMA: Neighbourhood built environment and children play spaces

Children often use open spaces that are available within their homes and neighborhoods to enhance their play and some of such spaces include inside the home, in gardens, school parks, undeveloped and unoccupied lands owned by private individuals and organizations, streets and pavements, religious open grounds (church/mosque) and large gutters. With about 31.3 percent out of the total population of GAMA (4,010,054) being children aged 0-14 (GSS, 2012), it is not surprising that children tend to find their own play space in their neighbourhood because they are also important stakeholders or users of the neighbourhood. It is however interesting to know that, these outdoor spaces within neighbourhoods are not being used only by children but also used by teenagers and adults. This is because the open spaces are used for multiple functions like playing, hawking or selling, and gathering for social (i.e., wedding, funeral, or naming ceremony) or religious activities.

**Figure 1:**
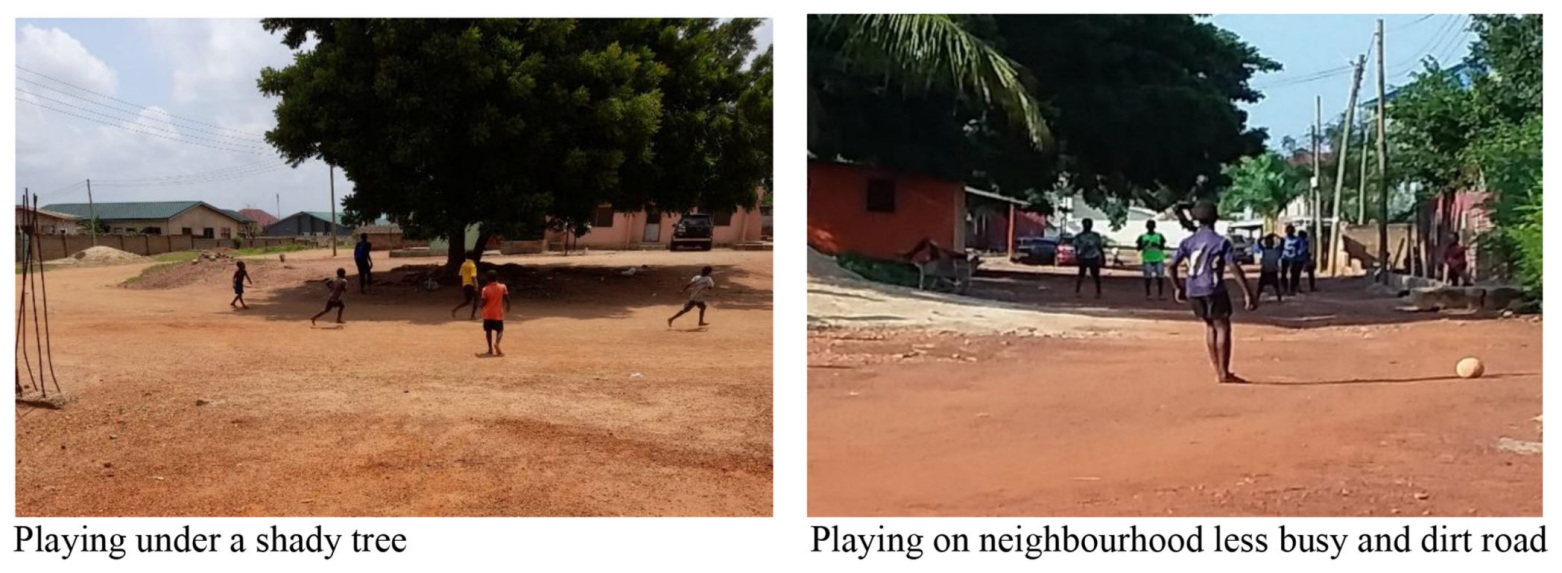
Pictures of informal outdoor play spaces

**Figure 2:**
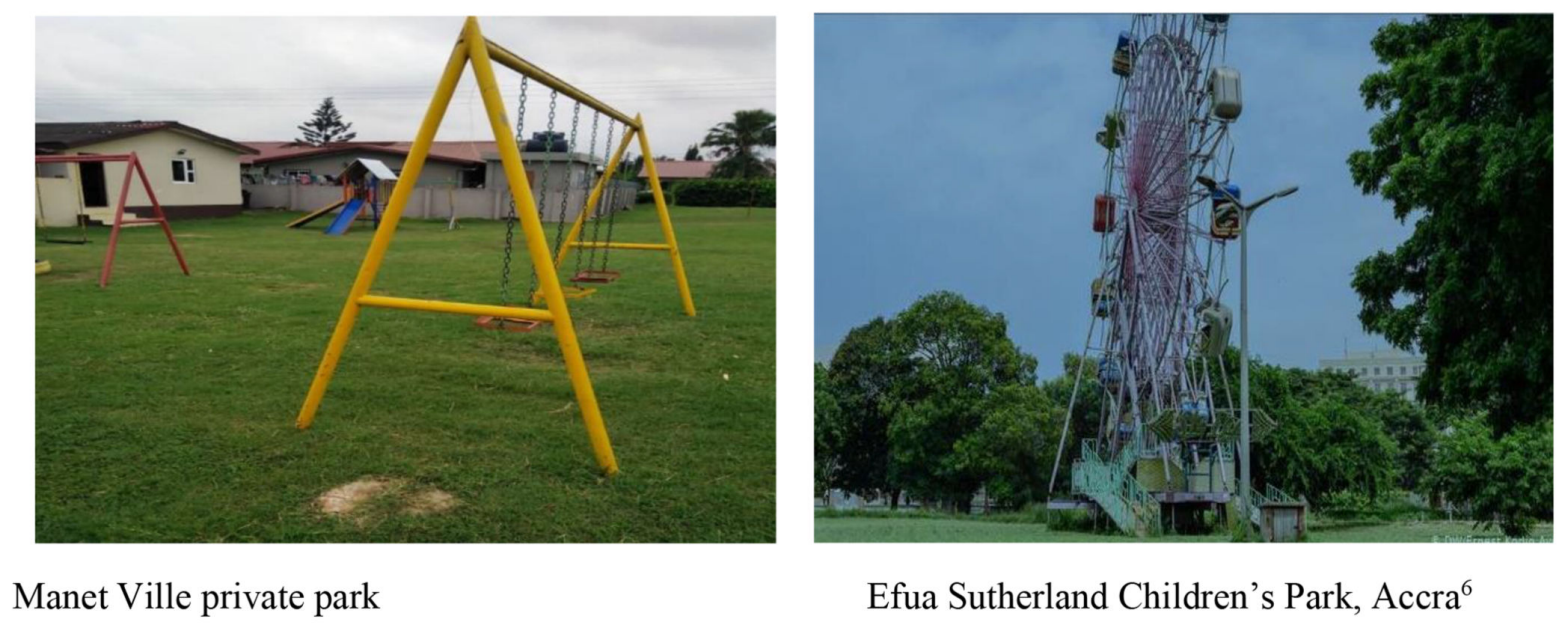
Pictures of formal outdoor play spaces

In broad terms, the playability of spaces within GAMA neighbourhoods can be classified into formal and informal play areas (see Table 5, and Figures 1 and 2). We defined formal play spaces as those specifically developed for a range of play activities with the approval of local governments. These include school parks, community playgrounds, open and green spaces, etc. On the other hand, informal play spaces refer to all child play spaces which are not approved and sanctioned by MMAs. Such spaces include a lot of spaces both large and small used by children to engage in play activities as indicated in Table 4.

Beyond the characterization, as local government approved or otherwise, the formal and informal play spaces differ by way of entry restrictions, distance from home, and security and safety. While informal play spaces may not possess any entry restrictions and are likely to be closer to homes or they may also be located in areas that may be described as high risk in terms of safety and security. Conversely, formal play spaces may be of higher quality but less accessible. For instance, it was reported recently in a news article that residents in Tema Metropolitan Assembly in GAMA were dissatisfied with the locking up of a community park redeveloped as Astro turfs. This is because these Astro turfs have been fenced with metal wires and put under lock and key, and thereby denying residents’ access and the use of the facility.^7^ Manyani et al. (2021) in their study of public green spaces in two South African towns concluded that the current design and available features within the studied spaces do not meet local preferences and needs as such communities benefit less from these spaces.

In addition, informal play spaces tend to be non-permanent spaces for children as many of these spaces are awaiting development by their owners. For instance, within GAMA undeveloped land and unoccupied residential plots are the most popular informal spaces for children play but the high demand for land for housing and other uses in the metropolitan region implies that the continuous use of these play spaces is not guaranteed. In other words, because informal play spaces for children have not been developed as approved spaces by local governments they are ad hoc, and their continuous use is hardly guaranteed.

Indeed, it needs to be stressed that it is the absence or limited access to formal play spaces which leads children to find and develop informal play places within their communities or neighbourhoods. Consequently, children, especially those in middle or low-income neighborhoods are more likely to spend more time in and around their neighbourhoods in informal open spaces and unplanned play spaces because planned formal play spaces are non-existent in these communities. Even formal children play facilities such as community playgrounds, open spaces and green spaces in urban Ghana can be sites for encroachment and contestations, especially in a metropolitan region such as GAMA (see Arku et al., 2016; Amoako & Adom-Asamoah, 2017).

Equally concerning and serving as a deterrent to outdoor play activities is poor waste management in many communities and neighbourhoods in GAMA. According to Owusu (2010) and World Bank (2015), although poor waste management affects all major cities in Ghana, the situation is direr in the GAMA due to high population density and congestion and weak governance and institutional structures. It has been observed that neighbourhoods with rubbish and filth, and poor sanitation in general, deter children from playing outdoors and this is because children are sensitive to their environment (UNFPA, 2007; Bartlett, 1999). As Bartlett (1999) and Hertzman et al., (2010) notes, poor living environments have particularly more negative far-reaching consequences for children than adults, as experiences and exposures during important developmental periods can have long-term impacts on health and wellbeing. Writing on Sabon Zongo, a poor neighbourhood in Accra, Owusu (2010) argued that the poor sanitation in the neighbourhood had adverse social consequences for children including impeding recreation and social learning of cultural rules and norms.

In the absence of proactive policies that place emphasis on children play spaces within urban Ghana, spaces for children’s play activities in GAMA undergoing rapid urbanization and intense demand for land, are under constant threat of encroachment and evictions. The common sight of children playing at hazardous locations such as large gutters, dumpsites, pedestrian walkways/road sidewalks, and on roads where they compete with vehicular traffic at the peril of their lives in the GAMA, especially in low-income neighbourhoods, underscores the challenges they face in securing play spaces. In this situation, many children are restricted to indoor play activities with its attendant negative consequences such as – increased sedentary behavior, low physical activity, mental health risks and lack of exposure to nature (Gray, 2011; Gray et al., 2015). Evidence suggests that under conditions of limited and unsafe play spaces within communities, children’s movements are restricted by parents and reduced to their immediate environs so that parents/guardians can keep an eye on the children while they play (Valentine, 1997; Chawla & Malone, 2003). Limiting children’s access to play, movement and social interaction by parents can have negative impacts on their health and overall growth and development (Mikkelsen & Christensen, 2009; Shaw et al., 2012).

## Conclusion

Most urban planning policies in Ghana focused on providing good roads, water, sanitation, and security in the community however, no central government or municipal policies directly address children’s need for play and recreation. Our policy document analysis demonstrates the limited policy on play in urban Ghana, especially GAMA, Ghana’s largest and densest metropolitan region. This is against the backdrop of a weak institutional framework for promoting inclusive and sustainable urban development. All projections indicate that the process of urbanization in Ghana and more especially GAMA will continue to intensify and is unlikely to slow until after 2040 (Owusu & Oteng-Ababio, 2015). This implies the likelihood of worsening of the current urban development challenges unless conscious efforts are made to address them. Again, evidence suggests a growing incidence of sedentary lifestyles, less physical activity, and the increasing health challenges associated with obesity among children and the youth in Ghana (Aryeetey, 2011; Ocansey et al., 2014; Seidu et al., 2020).

Nevertheless, the literature suggests that the availability of playable spaces within a built environment can go a long way to increase the variety of play opportunities that are offered to children and this helps create a more child-friendly place to live. (Gill, 2021)^8^ Such spaces are very important to the health and wellbeing of children since it affects their physical activity and ability to socialize with other children within the neighbourhood. Playable spaces are free from hazards and afford opportunities for engaging in active, social or focused play. Of course, it may have fixed facilities (e.g. play equipment) however, this is not always necessary - sometimes the natural environment or other people provide all the stimulation needed to play. It is therefore very important to know where children play, how they play, and where they intend to play in the future and whether their neighbourhood provides them with good and excellent play opportunities for the wellbeing of the child. According to Ledermann (1958, 37), “it is impossible for the child to discover the city unless the city discovers the child. The discovery must be reciprocal or there is no discovery at all”.

In considering play spaces for children, policymakers should not only take into account planned playgrounds but also consider the importance of unplanned spaces within the built environment. Children are always exploring the neighbourhood around them to suit their play needs irrespective of the spaces’ intended use. Careful consideration must therefore be made to create urban environments conducive to play. Playable space should be accessible, spacious, safe from physical and social hazards, allow healthy movement behaviors, provide opportunities for physical challenge, social interaction and exposure to nature (Garau et al., 2016; Voce, 2018). In the context of rapid urbanization in Ghana, cities have a unique opportunity to invest in sustainable environments and infrastructure that promote urban health and wellbeing, with impacts on Ghana’s population for years to come. Consequently, there is an urgent need to revise existing policy documents such as the National Urban Policy Framework & Action Plan, 2012, which is currently undergoing a review, to include specific policy goals and objectives to promote spaces for children’s play and recreation. Finally, Ghana should put a play policy together for children just as developed nations like England, Wales, and Australia have done, and in recent times in the Africa context as done by South Africa.

## Figures and Tables

**Table 1:** Gender patterns of play observed in Ghana

Gender	Type of outdoor activity	Play description
Male	Football	Most popular outdoor play. Though normally played with 11 players on both sides, many children tend to improvise with any desirable number usually based on space and children available.
	‘Pilolo’ and ‘Pampanaa’ (Hide and seek)	These two games develop the navigational skills of the child since it involves hiding from each other and mostly played in uncompleted buildings. It is played in groups.
	‘Chaskele’ (Ghanaian kids’ version of cricket)	Played with a stick and empty milk cans and a car tyre. The car tyre is placed on the ground and players try to toss the can into the hole in the tyre while other players try to prevent the can from entering into the hole by hitting the can away.
Female	Skipping rope	A play where one or two kids jump over a rope being swung by two other kids
	‘Ampe’	This game involves jumping, singing and clapping and it is played by two or more people. The number is always even
	‘Tumatu’ (Ghanaian version of hopscotch)	This game involves jumping and being strategic. You have to put your opponent in a position that makes it difficult or impossible to jump

**Table 2:** Selected key policy documents in Ghana reviewed and assessed

Policy	Relevant Policy Goal/Strategy	Policy on children	Policy includes ‘play’	Policy includes ‘recreation’
National Youth Policy, 2010	Promote youth participation in sports, recreation, and positive leisure activities, including the provision of playgrounds	✓	X	✓
National Urban Policy Framework & Action Plan, 2012	Among policy measures to protect open spaces and ecologically sensitive areas include “develop and use open spaces, green belts and other ecologically sensitive areas (i) for appropriate recreation and urban farming; (ii) to enhance visual amenity; and (iii) to promote microclimate control as appropriate.	X	X	✓
National Housing Policy, 2015	Mandate the identification and acquisition of open spaces for active and passive public recreation in neighbourhoods and communities.	X	X	✓
Child and Family Welfare Policy, 2015	Policy establishes a coordinated child and family welfare system that promotes the wellbeing of children, prevents abuse and protects children from harm. No explicit or implicit policy objective on playability	✓	X	X
National Spatial Development Framework, 2015-2035	Overall spatial development strategy includes green infrastructure network (GIN) in urban areas such as outdoor recreational and educational facilities, promoting community interaction with the environment to improve physical and social inclusion for the particular benefit of the young, disabled and older segments of the populations	X	X	✓
Children’s (Amended) Act (Act 937), 2016	Article 9 – Right to Social Activity. No person shall deprive a child the right to participate in sports, or in positive cultural and artistic activities or other leisure activities.	✓	X	✓
Ghana Building Code, 2018	Provides guidelines on public open spaces such as parks and gardens, small play areas within shopping malls, and other open areas for children	✓	✓	✓
National Medium-Term Development Policy Framework (NMTDPF) - An Agenda for Jobs: Creating Prosperity and Equal Opportunity for All, 2018-2021	Integrate sports and recreational needs of the aged and children in the provision of facilities (SDG Target 11.7)	✓	X	✓
Medium-term development plans of municipalities, 2018-2021	Localization of the policy objectives of the NMTDPF - An Agenda for Jobs: Creating Prosperity and Equal Opportunity for All, 2018-2021, at the city/municipal level	✓	X	✓
Revised National Health Policy, 2020	Support the development of recreational and physical activity facilities for use by the population regularly to achieve longterm health.	X	X	✓

*Note:* (✓) indicates policy has measures on children, play/playability or recreation while (x) means otherwise.

**Table 3:** Grades according to physical activity indicator in the Ghana Report Card on Physical Activity for Children and Youth, 2014, 2016 and 2018

Indicator	Grade		
	2014	2016	2018
Overall Physical Activity Levels	D	D	C
Organized Sport Participation	C	C	C+
Active Play	INC	B	B-
Active Transportation	D	C	C+
Sedentary Behaviors	B	D	INC
Family and Peers	INC	F	F
School infrastructure, policies and programs	D	D	D
Community and the Built Environment	D	F	D+
Government Strategies and Investments	D	D	D

*Note:* The grade for each indicator is based on the percentage of children and youth meeting a defined benchmark: A is 81%–100%; B is 61%–80%; C is 41%–60%, D is 21%–40%; F is 0%–20%; INC is incomplete/Inconclusive owing to insufficient data.

Note. “+” and “-” signs are added to the grades in some circumstances to indicate the high or low end of the grade continuum respectively and/or to indicate the presence (“-”) or absence (“+”) of significant gender, geographic, ethnic, or socioeconomic disparities.

Source: Ocansey et al. (2014, 59; 2016, 166), Nyawornota (2018, 367)

**Table 4:** Global School-based Student Health Survey 2007 and 2012 for children aged 13-15 years

Physical Activity	Total		Boys		Girls	
	2007	2012	2007	2012	2007	2012
Percentage of students who were physically active for a total of at least 60 minutes per day during the past 7 days	16.3	16.0	16.8	15.9	16.1	16.1
Percentage of students who went to physical education (PE) class on three or more days each week during this school year*	N/A	32.2	NA	33.7	N/A	31.0
Percentage of students who spent three or more hours per day during a typical or usual day sitting and watching television, playing computer games, talking with friends, or doing other sitting activities	23.7	19.1	24.8	18.0	22.6	20.0

* Physical Education was not captured in 2007 Source: WHO and Centers for Disease Control and Prevention. Ghana Global School-based Student Health Survey (GSHS). http://www.cdc.gov/gshs/

**Table 5:** Formal and informal children play spaces in Accra

Formal play space	Informal play space
School parks Community playgrounds Open spaces Green spaces Shopping mall playgrounds Sport stadiums Community Astro turfs	Undeveloped lands Unoccupied residential plots Religious grounds Less busy roads Back alleys Large gutters/drains Pedestrian walkways/road sidewalks Home courtyards Dumpsites

## Highlights

- Limited research on children’s play in Africa in general and Ghana in particular.
- ‘Play’ and ‘playability’ feature less in policy documents.
- Efforts to promote physical activities focused on walkability, less on playability.
- Weak city planning limits opportunities for children outdoor spaces.
- Change in policy on children outdoor play space in urban Ghana needed.

## Footnotes

1 A child in this paper is defined “as a person below the age of 18 in line with the UN Convention on the Rights of the Child, the 1992 Fourth Republic Constitution of Ghana (Article 28) and the Children’s Act 1998 (Act 560)” (GoG/MC&SP, 2014, iv).

2 Committee on the Rights of the Child. (2013). General Comment No.17 (2013) on the right of the child to rest, leisure, play, recreational activities, cultural life and the arts (Article 31).

3 Although cul-de-sacs offer opportunities for children outdoor play, they may also be associated with lower walkability for adults (due to lower connectivity, lower density) and higher dependence on car-based transportation. Nevertheless, cul-de-sacs offer quiet and safe streets where children can play without fear of traffic, and residents are more likely to know their neighbours, which is also important for safety.

4 In this paper, we see children outdoor play as self-motivated and intrinsic, and naturally as part of the emotional, psychological and physical growth process of children, and not as an activity undertaken during their free time.

5 In Ghana, there are three types of local governments: District Assemblies (with population over 75,000 and largely rural with small/medium-sized towns as administrative capital); Municipal Assemblies (contiguous large towns or urban centres with population over 95,000) and; Metropolitan Assemblies (large cities/metropolitan centres with population over 250,000).

7 Make open parks accessible to residents - Tema residents charge. https://www.ghanaweb.com/GhanaHomePage/regional/Make-open-parks-accessible-to-residents-Tema-residents-charge-1111927. Accessed on May 16, 2021.

8 What is playable space? https://inspiredplay.com/what-is-playable-space/. Accessed on May 17, 2021.
